# Cognitive neural responses in the semantic comprehension of sound symbolic words and pseudowords

**DOI:** 10.3389/fnhum.2023.1208572

**Published:** 2023-10-11

**Authors:** Kaori Sasaki, Seiichi Kadowaki, Junya Iwasaki, Marta Pijanowska, Hidehiko Okamoto

**Affiliations:** ^1^Department of Speech and Hearing Sciences, International University of Health and Welfare, Narita, Japan; ^2^Graduate School of Medicine, International University of Health and Welfare, Narita, Japan; ^3^Office of Medical Education, International University of Health and Welfare, School of Medicine, Narita, Japan; ^4^Graduate School of Humanities and Sociology, University of Tokyo, Tokyo, Japan

**Keywords:** semantic comprehension, sound symbolism, onomatopoeia, event-related potential, lexical semantic processing

## Abstract

**Introduction:**

Sound symbolism is the phenomenon of sounds having non-arbitrary meaning, and it has been demonstrated that pseudowords with sound symbolic elements have similar meaning to lexical words. It is unclear how the impression given by the sound symbolic elements is semantically processed, in contrast to lexical words with definite meanings. In event-related potential (ERP) studies, phonological mapping negativity (PMN) and N400 are often used as measures of phonological and semantic processing, respectively. Therefore, in this study, we analyze PMN and N400 to clarify the differences between existing sound symbolic words (onomatopoeia or ideophones) and pseudowords in terms of semantic and phonological processing.

**Methods:**

An existing sound symbolic word and pseudowords were presented as an auditory stimulus in combination with a picture of an event, and PMN and N400 were measured while the subjects determined whether the sound stimuli and pictures match or mismatch.

**Results:**

In both the existing word and pseudoword tasks, the amplitude of PMN and N400 increased when the picture of an event and the speech sound did not match. Additionally, compared to the existing words, the pseudowords elicited a greater amplitude for PMN and N400. In addition, PMN latency was delayed in the mismatch condition relative to the match condition for both existing sound symbolic words and pseudowords.

**Discussion:**

We concluded that established sound symbolic words and sound symbolic pseudowords undergo similar semantic processing. This finding suggests that sound symbolism pseudowords are not judged on a simple impression level (e.g., spiky/round) or activated by other words with similar spellings (phonological structures) in the lexicon, but are judged on a similar contextual basis as actual words.

## 1. Introduction

The arbitrary relationship between form and meaning is one of the essential characteristics of language (Hockett, 1960). Language is said to be arbitrary as there is usually no special reason why a specific form (sound or shape) is used to express a certain meaning. For example, different words, such as “apple” in English and “リンゴ (ringo)” in Japanese, are used to convey the same idea. An exception to this rule, however, is onomatopoeia. Onomatopoeia is the imitation of an actual sound that has become a standard lexical item (e.g., “bow wow” for a dog’s bark). As such, onomatopoeia is considered a lexeme with a non-arbitrary connection between form and meaning (Pinker, 1999). This kind of non-arbitrary association of sound and meaning is known as sound symbolism. Sound symbolism is defined as “the direct link between sound and meaning” (Hinton et al., 1994).

Sound symbolism has been demonstrated in experiments in which participants consistently associate certain types of phonemes with specific shapes. The phoneme is the smallest unit of sound used in the word. For instance, plosives such as /t/ and /k/ are often perceived as referring to a straight line or sharp-edged figure, and nasals like /m/,/n/ as referring to a curved line or rounded figure (Köhler, 1947; Ramachandran and Hubbard, 2001). It is believed that the sound symbolism of some phonemes and phoneme clusters is connected to the structural characteristics of the brain or the mechanism by which information is processed in the brain (Sidhu and Pexman, 2018).

While many such sound symbolic elements are consistently associated with certain meanings, they are not usually considered a part of the mental lexicon. The mental lexicon is a set of words that humans retain in their brains, the idea behind a mental lexicon is that the vast majority of information related to a word is stored in long-term memory as a concept (Aitchison, 2012). Usually, using this mental lexicon is necessary to comprehend a word’s meaning (Stille et al., 2020). Words are encodings of a specific object, such as an event, concept, or thing, with a linguistic label (Abe et al., 1994). Moreover, each word has a distinct pronunciation, spelling, meaning, and syntactic category, such as noun or verb, which is stored in a mental lexicon. In contrast, sound symbolic elements are not associated with any syntactic category and their semantic content is much vaguer compared to standard lexical items–they only provide impressions, such as round, soft, hard, or sharp (Ramachandran and Hubbard, 2001).

On the other hand, established sound symbolic words, such as onomatopoeia, can be considered full-fledged lexical items. The inventory of such words in Indo-European languages is mostly limited to onomatopoeia that express the sounds made by animals (ex., quack-quack) or sounds made while using certain objects (boom or swish). However, many languages outside of the Indo-European family use sound symbolic words known as ideophones that can describe manners, states and emotions (Osaka, 1999; Akita, 2017). In this study, we focus on Japanese, in which a great variety of such sound symbolic words is frequently used. Japanese onomatopoeia and ideophones both usually incorporate sound symbolic elements with consistent semantic associations (ex./k/: gives the image of hitting a hard surface,/g/: gives the impression of increased strength/weight) (Hamano, 2014), and use identical morphological patterns (often a reduplication of 2-mora elements, as in/fuwa-fuwa/) (Tamori and Schourup, 1999). Onomatopoeia and ideophones are established items of Japanese mental lexicon and in this study, we use the term sound symbolic words to refer to both of these categories.

Phonological mapping negativity (PMN) and the N400 response of event-related potentials (ERPs) have been used as indicators in studies of the processing mechanisms of words and pseudowords. A negative evoked brain response connected to language processing, known as PMN, which has an earlier latency than N400, manifests around between 250 and 300 ms (Desroches et al., 2009). According to van den Brink et al. (2001), PMN represents the stage at which, in relation to the lexical selection process, word-form information resulting from an initial phonological analysis and content information derived from the context interact. However, Newman et al. (2003), and Newman and Connolly (2009) discovered that PMN occurs during the preliminary stage of language processing and reflects phonological processing. Thus, some reports of PMN indicate that it is involved in higher-level language processing, while others indicate that it is only involved in phonological processing, which occurs earlier. The interpretation of PMN depends on whether the task focuses solely on the phonological aspect of the word or on determining if the word is an appropriate choice based on higher-level linguistic characteristics.

N400 is a significant negative wave that peaks around 400 ms after the stimulus is presented (Kutas and Federmeier, 2011). The amplitude of N400 has been proven to increase for sentences with mismatched meanings (e.g., “I like coffee with cream and socks”) (Kutas and Hillyard, 1980) and is considered to be a brain potential response associated with semantic processing. Some studies of N400 interpret it as reflecting the process of retrieving words from the mental lexicon, whereas others interpret it as reflecting higher-order processes, like context specific semantic processing of vocabulary. In the former interpretation, Petten and Kutas (1990) showed that the amplitude of N400 increases for low frequency words compared to high frequency words, and Kutas and Federmeier (2000) stated that the N400 reflects the activation of vocabulary stored in long-term memory. These studies indicate that N400 may reflect access to the mental lexicon. On the other hand, Brown and Hagoort (1993) found that N400 reflects not only the process of accessing the mental lexicon but also the higher order semantic processing, as N400 is smaller when the priming effect is involved. Hagoort et al. (2004) also reported that both accessing the mental lexicon and integration into semantic knowledge happened at the same time, beginning 300 ms after language presentation. They reported that N400 is caused by the top-down influence of semantic information. In previous studies, the interpretation of PMN and N400 depended on the nature of the task and stimulus words. In their report of N400 in sound symbolic pseudowords, Deacon et al. (2004) showed that non-words derived from existing words show the same level of semantic activation as existing words when participants listen to them. On the other hand, Westbury (2005), in a study using sound symbolic pseudowords, reported that the lexical access stage, which is a preliminary stage of semantic processing, is activated, indicating that the effect of sound symbols is pre-semantic.

In past studies, the interpretation of PMN and N400 was dependent on the nature of the task and stimulus words. Moreover, previous studies on the sound symbolism of pseudowords mostly involved judgment tasks that use simple pictures of round or angular shapes with no deeper meaning (Kovic et al., 2010; Asano et al., 2015; Sučević et al., 2015). In contrast, the task setting in this study was designed to reflect semantic processing by asking the participants to determine whether the depicted situation or object and matched or mismatched the sound stimuli (existing sound symbolic words or pseudowords). In the sound symbolic word task, there is always a correct word for the situation. On the other hand, the sound-symbolic pseudoword task is a non-word that does not correspond to any of the tasks. Sound symbolic pseudowords are non-existing words created for this study and, unlike established onomatopoeia and ideophones, cannot be considered part of the mental lexicon. Based on previous reports, it is possible that the processing of sound symbolic pseudowords also involves semantic processing; however, it is not clear whether the process is similar to that of established lexemes contained in the mental lexicon.

The goal of this study is to investigate the differences between the semantic processing of established sound symbolic words and pseudowords. Based on the results of previous studies, we suppose that the amplitude of both the PMN and the N400 will be larger in the mismatch condition than in the match condition when assessment of whether the language is consistent with more complex situations requires semantic processing. If so, the PMN appears to reflect neural processing that includes not only phonological processing, which is the precursor to language processing, but also linguistic processing (e.g., word selection). The phonological structure of sound symbolic pseudowords used the common patterns observed in existing Japanese sound symbolic words, and it included sound symbolic phonemes typical in Japanese. The phonological structure of sound symbolic pseudowords may provoke semantic neural processing. Therefore, it would support the hypothesis that sound symbolic pseudowords are semantically processed in the same way as the words if the amplitude of both the PMN and the N400 increased more in the mismatch condition than in the match condition in the sound symbolic pseudoword task, similarly to the sound symbolic words.

In the present study, we aim to clarify the processes reflected by PMN and N400 and to identify the differences in the semantic processing for existing sound symbolic words and sound symbolic pseudowords. The results obtained will give us some clues regarding the neural process of phonological and linguistic features of sound symbolism.

## 2. Materials and methods

Event-related potentials (ERPs) were measured and analyzed while sound symbolic word and pseudoword comprehension tasks were carried out in order to investigate the specifics of neural processing during lexical processing when a picture of an event and a speech sound are consistent (match) or inconsistent (mismatch).

### 2.1. Participants

A total of 30 healthy university students, ranging in age from 19 to 22, took part in the study. Participants were paid volunteers recruited at the International University of Health and Welfare. They were all native Japanese speakers and had no medical or mental health issues in the past. They had normal or corrected-to-normal vision, and they had normal hearing. The International University of Health and Welfare’s Ethical Review Committee gave its approval to this study. The experiment was explained to participants orally and in writing, and they voluntarily signed an informed consent form. This study was conducted in accordance with the ethical principles of the Declaration of Helsinki. Three participants were excluded from the study because their correct response rate to the task was less than 80%, and seven participants were excluded because the difference between their correct response rate to the sound symbolic word task and the sound symbolic pseudo-word task was more than 5%. In addition, two had many artifacts (less than 80% valid epochs) and one had incomplete task results. We used data from a total of 17 subjects for the final analysis.

### 2.2. Stimulus

In both sound symbolic word and pseudoword tasks, pictures and sounds were presented. In both task types, match conditions—where the appropriate sound for the picture was presented—and mismatch conditions—where a sound unrelated to the picture was presented—were used.

There were 160 stimulus word sounds, 80 each of sound symbolic words and pseudowords. The Japanese Onomatopoeia Dictionary (Ono, 2007) was used to choose existing sound symbolic words. The most prevalent type, the two-mora reduplication sound symbolic word (e.g., /fuwafuwa/, meaning “soft and puffy”) (Tamori and Schourup, 1999), was used as the sound stimulus. We chose words that expresses various receptive senses like touch and hearing, and the meaning is relatively clear and not context-dependent. Since sound symbolic words used in Japanese to express feelings often change their meaning depending on the situation (like/moyamoya/: thoughts and memories to be fuzzy, but also the feeling of unease or pent-up lust, depending on context), we limited our stimuli to sound symbolic words that express clear sensory states or manners. Established sound symbolic words that were used as stimulus words were modified to create sound symbolic pseudowords. The first and third syllables were not altered during creation; the second and fourth syllable were. Additionally, in keeping with Hamano’s (2014) research, we changed the second syllable to a sound symbolic phoneme that elicits the same semantic impression, for example, roundness or sharpness, to preserve the word’s overall semantic characteristics (e.g., /fuwafuwa/versus/fuhafuha/) (Supplementary Data Sheet 1).

A Text to speech program (Azure, Text to speech, Microsoft Inc., Redmond, WA, USA) was used to synthesize the voice of the stimulus words. All stimulus words were controlled using version: 1.0, voice type: male voice, prosody rate: 50%, pitch: 0%, used language: Japanese. We manipulated the reading speed (between 0.5 and 1.5) to fit the length of a sample to around 500 ms. In order to use the generated speech samples as stimuli for the task, the length of the speech sample was then adjusted to exactly 500 ms in Audacity 3.2, an open-source audio file editing program.^1^ Fade in/out effects were added to the first and last 10 ms of each speech sample.

**FIGURE 1:**
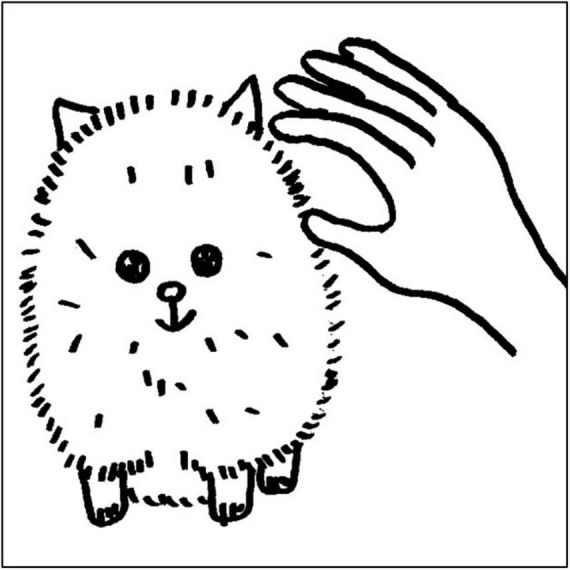
An example of stimulus illustration. Existing Japanese sound symbolic word–onomatopoeia:/fuwa fuwa/, which means “fluffy” in English, the corresponding sound symbolic pseudoword:/fuha fuha/. In this illustration,/fuwa- fuwa/indicates the dog’s hair is soft.

Both the sound symbolic word and pseudoword tasks made use of the same stimulus pictures. The meaning of established lexemes was verified in a Japanese onomatopoeia dictionary, and corresponding black-and-white pictures were created to act as the stimulus pictures (Figure 1).

There were 160 conventional sound symbolic word tasks, 80 of which were in the match condition and contained the appropriate stimulus word and picture combination. The other 80 tasks were in the mismatch condition and contained the inappropriate stimulus word and picture combination. The sound symbolic pseudoword tasks were created by replacing the existing word with a corresponding pseudoword with alternate phonemes for both the match and mismatch conditions. Consequently, there were 160 sound symbolic pseudoword tasks, 80 of which were in the match condition and 80 in the mismatch condition. Both sound-symbolic words and sound symbolic pseudowords tasks are presented twice for 40 words, for a total of 80 tasks. All of the auditory and visual stimuli were the same in the match and mismatch conditions but in different combination. The sound symbolic word and pseudoword tasks’ match and mismatch conditions added up to 320 tasks in total. In the sound symbolic word and sound symbolic pseudoword tasks, the stimulus words were presented randomly in each task.

### 2.3. Procedure

Participants in the experiment were seated in a soundproofed, electrically shielded room. While the participants were deciding whether the picture on the display matched the sound coming from their earphones, their brain waves were monitored. Button-pushing tasks were given to participants to ensure they were paying close attention during the experiment. The participants were monitored while the tasks were carried out to make sure the participant was paying attention. Participants practiced on several words in both the sound symbolic task and pseudoword task prior to the actual experiment. Participants began the actual experiment when they understood the content of the task.

**FIGURE 2:**
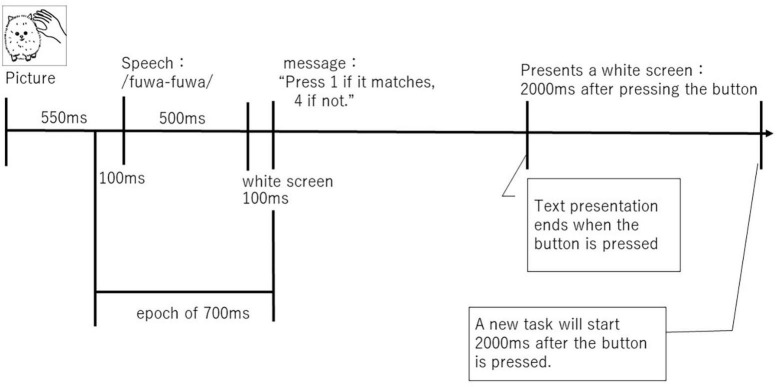
A schematic figure of audio-visual stimulation.

The experimental design is schematically represented in Figure 2. Stimulus pictures were shown on a 23.0″ LCD screen with a resolution of 629 × 629 pixels (FlexScan EV2316, EIZO Inc., CA, USA, JP, resolution: 1,920 × 1,080), Subject-to-screen distance: range 50–85 cm, average 70 cm, and audio was played through ER-3A insert earphones (Etymotic Research Inc., IL, USA). The experiment started with the display of a stimulus picture. Next, 550 ms after the stimulus picture was displayed, a stimulus word sound (sound symbolic words or pseudowords) was presented for 500 ms. After the audio presentation, a white screen was displayed for 100 ms. Following this, a message prompting the user to interact (“Press 1 for a match, 4 for a mismatch”) was displayed in MS Gothic font size 80. Participants immediately responded by pressing the 1 or 4 key on the keyboard. The message disappeared once the participant chose a response. The next task started after 2,000 ms of a white screen. The Medical Try System Multi Trigger System Ver. 2 (MTS0410, Medical Try System, Co., Ltd., Japan) was used to control the task presentation, and the match and mismatch conditions were distributed at random.

During the experiment, participants were told to avoid blinking, swallowing saliva, or unnecessary movement. In addition, the sound symbolic word and sound symbolic pseudoword tasks were counterbalanced by randomly administering the sound symbolic word and sound symbolic pseudoword tasks to each subject. A break was always included between the pseudoword task segment and the existing word task segment. Additionally, breaks were also taken during the segments upon the participant’s request. The experiment took approximately 30–40 min to complete.

### 2.4. Acquiring EEG data

A unipolar recording of EEG signals was made at the Cz position. Because PMN and N400 are greatest at the parietal site (Dumay et al., 2001: D’Arcy et al., 2004; Kutas and Federmeier, 2011), PMN and N400 were measured at Cz in this experiment. Electrodes placed on the mastoids served as a reference, and the grounding electrode was positioned on the forehead. EEG signals were captured on a Neurofax EEG-1200 system (Nihon Koden, Co., Ltd., Japan). Before starting the task, it was determined that the contact impedance between the skin and electrode was less than 10 kΩ. The signal was converted to a digital signal at 1 kHz after being bandpass filtered between 0.3 and 30 Hz. The data were divided into epochs of 700 ms, after the speech stimulus presented at 100 ms. The baseline was set based on the 100 ms prior to the presentation of the speech stimulus. Reactions associated with blinking, eye movements, etc. were rejected offline based on electrooculography. Upon visual inspection, epochs with excessive noise were removed from the analysis. The total number of data points in each condition averaged 69.7 epochs (range: 79–65).

### 2.5. Statistical analysis

In this study, PMN and N400 components are of particular interest. Therefore, the maximum amplitude between 250 and 350 ms after the sound onset was defined as PMN amplitude (Lee et al., 2012) and its latency was defined as PMN latency. The average amplitude between 350 and 500 ms after the sound onset was defined as N400 amplitude (D’Arcy et al., 2004; Asano et al., 2015; Manfredi et al., 2017). We analyzed PMN and N400 using Matlab R2022b (MathWorks, Inc., USA). For the existing word and pseudoword tasks, as well as for each of the amplitudes and emergent latencies, PMN was subjected to a two-way ANOVA (two factors: existing word task/pseudoword task x match/mismatch). The N400 amplitude was similarly analyzed by a two-way ANOVA using sound type (existing word vs. pseudoword) and audio-visual matching (match vs. mismatch) as factors. The statistical significance for all two-way ANOVA was expressed as a *p*-value of less than 0.05. Effect size was calculated using partial eta squared (η*_*p*_*^2^) and generalized eta squared (η*_*G*_*^2^) (Bakeman, 2005).

In order to more clearly analyze the differences between sound symbolic words and pseudowords, planned comparisons were conducted using paired *t*-tests between matching and mismatching conditions in each existing word and pseudoword task, as well as between the existing word and pseudoword task in each matching and mismatching condition. Cohen’s *d* was used to examine effect size, with 0.2 small, 0.5 medium, and 0.8 large (Cohen, 1988). We used IBM SPSS Statistics Version 29 for Windows (IBM Corp., Armonk, NY, USA).

## 3. Results

### 3.1. Behavioral results

The average score for the sound symbolic word task for the participants was 151, and the average score percentage was 95%. On the other hand, the mean score for the sound-symbolic pseudoword task was 149, and the mean percentage score was 93%.

### 3.2. Electrophysiological results

**FIGURE 3:**
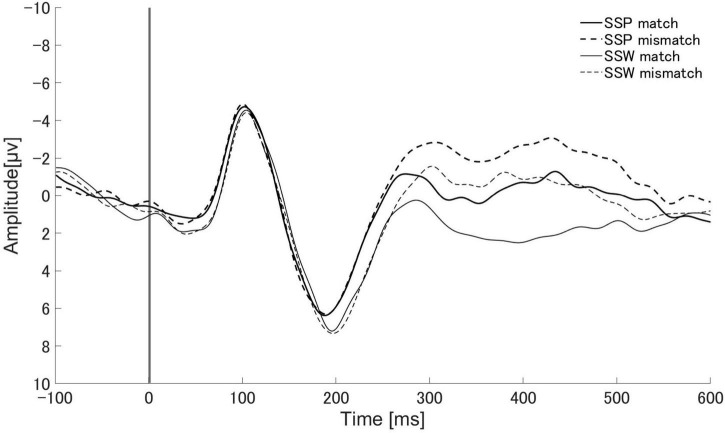
Auditory evoked responses elicited by sound symbolic words (SSW: gray lines) and sound symbolic pseudowords (SSP: black lines). Solid and dotted lines represent audio-visual matching and non-matching conditions, respectively. SSP match: match condition of sound symbolic pseudowords task. SSP mismatch: mismatch condition of sound symbolic pseudowords task. SSW match: match condition of sound symbolic words task. SSW mismatch: mismatch condition of sound symbolic words task.

Figure 3 displays the mean Cz brain wave reading for the match and mismatch conditions of established sound symbolic words and pseudowords from 100 ms prior to speech presentation to 600 ms following presentation.

A significant main effect of sound type (existing words vs. pseudoword) was observed with the maximum amplitude in the PMN (250–350 ms) component of the ERP [*F*_(1_, _16)_ = 11.89, *p* = 0.003, η*_*p*_*^2^ = 0.426, η*_*G*_*^2^ = 0.030] (Supplementary Data Sheet 2). The main effect of audio-visual matching (match vs. mismatch) was also observed [*F*_(1_, _16)_ = 10.07, *p* = 0.006, η*_*p*_*^2^ = 0.386, η*_*G*_*^2^ = 0.030]. No significant interaction was found between sound type and audio-visual matching [*F*_(1_, _16)_ = 0.21, *p* = 0.653, η*_*p*_*^2^ = 0.0013, η*_*G*_*^2^ < 0.001]. A significant main effect of audio-visual matching was observed for the latency at which the PMN peak appeared [*F*_(1_, _16)_ = 19.46, *p* < 0.001, η*_*p*_*^2^ = 0.549, η*_*G*_*^2^ = 0.085] (Supplementary Data Sheet 3). There was no significant difference in the sound type [*F*_(1_, _16)_ = 0.03, *p* = 0.875, η*_*p*_*^2^ = 0.002, η*_*G*_*^2^ < 0.001] and no significant interaction between sound type and audio-visual matching [*F*_(1_, _16)_ = 0.42, *p* = 0.529, η*_*p*_*^2^ = 0.025, η*_*G*_*^2^ = 0.002]. The mean amplitude in the N400 range (350–500 ms) showed significant main effects for sound type [*F*_(1_, _16)_ = 16.24, *p* = 0.001, η*_*p*_*^2^ = 0.504, η*_*G*_*^2^ = 0.064] and audio-visual matching [*F*_(1_, _16)_ = 36.68, *p* < 0.001, η*_*p*_*^2^ = 0.696, η*_*G*_*^2^ = 0.070] (Supplementary Data Sheet 4). There was no significant interaction between sound type and audiovisual agreement [*F*_(1_, _16)_ = 0.84, *p* = 0.374, η*_*p*_*^2^ = 0.050, η*_*G*_*^2^ = 0.002]. The amplitudes of PMN and N400 showed significant differences for sound type and audio-visual matching, but no significant interaction between them. The PMN latencies showed a significant difference for audio-visual matching, but no significant difference for sound type and no significant interaction between them.

**FIGURE 4:**
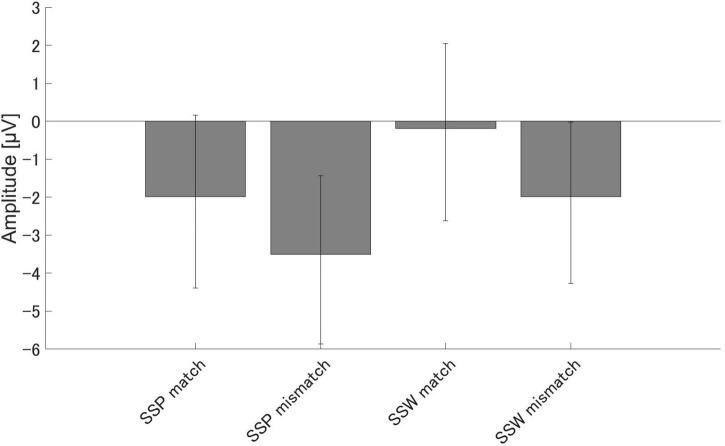
Group means (*N* = 17) of PMN amplitudes. Group means (*N* = 17) of PMN, the maximum amplitude elicited 250–350 ms after speech presentation. The error bars denote 95% confidence intervals. SSP, Sound symbolic pseudowords, SSW, sound symbolic words.

There were significant differences in the amplitudes of PMN between the match and mismatch conditions for the sound symbolic words [*t* (16) = 2.93, *p* = 0.010, Cohen’s *d* = 0.711] and pseudowords, [*t* (16) = 2.55, *p* = 0.021, *d* = 0.619]. Furthermore, there were significant PMN amplitude differences between the sound symbolic word and pseudoword tasks for the match condition [*t* (16) = 3.07, *p* = 0.007, *d* = 0.744] and mismatch conditions [*t* (16) = 2.73, *p* = 0.015, *d* = 0.663] (Figure 4).

**FIGURE 5:**
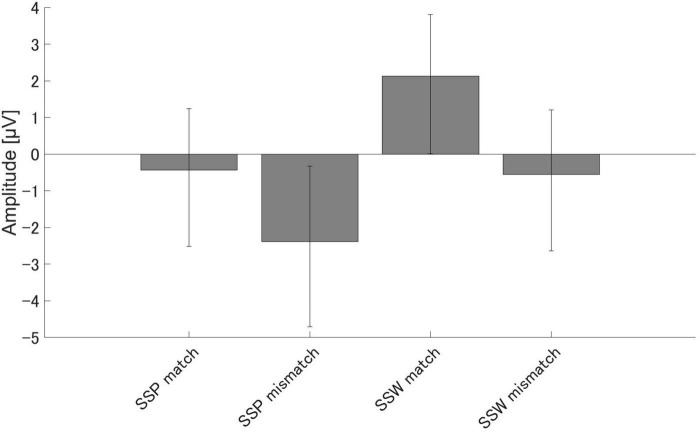
Group means (*N* = 17) of N400 amplitudes. Group means (*N* = 17) of N400 amplitudes elicited 350–500 ms after speech sound presentation. The error bars denote 95% confidence intervals.

Similar results were obtained for N400. We found significant N400 amplitude differences between the match and mismatch conditions for sound symbolic words [*t* (16) = 6.00, *p* < 0.001, *d* = 1.455] and pseudowords [*t* (16) = 2.87, *p* = 0.011, *d* = 0.697], and between the sound symbolic word and pseudoword tasks for the match [*t* (16) = 4.66, *p* < 0.001, *d* = 1.129] and mismatch conditions [*t* (16) = 2.25, *p* = 0.039, *d* = 0.546] (Figure 5).

**FIGURE 6:**
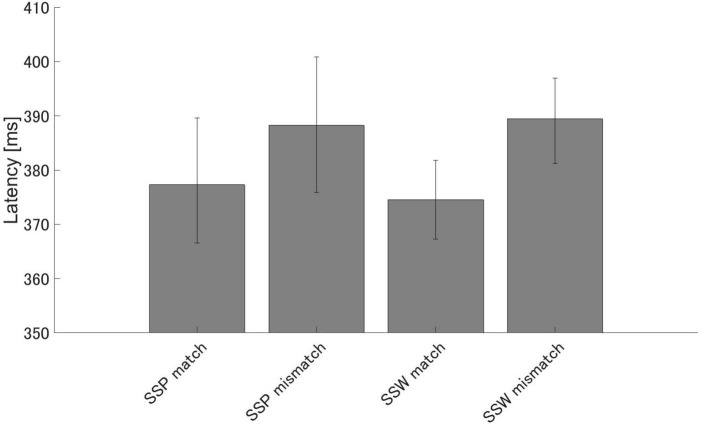
Group means (*N* = 17) of PMN latencies. Group means (*N* = 17) of PMN latencies that elicited maximum amplitude 250–350 ms after speech presentation. The error bars denote 95% confidence intervals. SSP: sound symbolic pseudowords, SSW: sound symbolic words.

There were significant differences in PMN latency between the match and mismatch conditions for sound symbolic words [*t* (16) = −3.64, *p* = 0.002, *d* = −0.882] and pseudowords [*t* (16) = −2.58, *p* = 0.020, *d* = −0.626], but no significant difference in PMN latency between the sound symbolic word and pseudoword tasks for the match [*t* (16) = −0.49, *p* = 0.632, *d* = −0.119] nor mismatch conditions [*t* (16) = 0.13, *p* = 0.902 *d* = 0.030] (Figure 6).

## 4. Discussion

This study investigated the semantic processing of both existing sound symbolic words and sound symbolic pseudowords with similar phonological structures using ERPs elicited while making semantic assessments of whether the sound stimuli are a match or mismatch with a picture of an event. During the task of determining whether pictures of events and speech sounds match or mismatch PMN and N400 were measured, and the semantic processing of sound symbolic words and pseudowords was examined and compared. The results confirm the hypothesis that PMN reflects not only phonological but also word processing, while N400 reflects contextual semantic processing. Furthermore, both the sound-symbolic pseudowords (non-words) and the sound-symbolic words (existing words) showed an amplitude increase in the mismatch condition compared to the match condition, suggesting that pseudowords, like existing words, may undergo semantic processing.

Connolly et al. (2001) discovered that when phonetic features match between the target word and a candidate word, less phonological processing work is needed, and the PMN is reduced; when they do not match, more thorough phonological analysis is needed, and a larger PMN is generated. This study presented pictures presented prior to sounds, so pictures are prime for target words. The PMN results of the present study confirmed the findings of Connolly et al. (2001) by demonstrating an increase in amplitude in the mismatch condition compared to the match condition for conventional sound symbolic words. Thus, the results of this study support the hypothesis of van den Brink et al. (2001) and Kujala et al. (2004) that PMNs reflect a continuum of processing at the acoustic, phonological, and word levels, suggesting that PMNs do not represent phonological processing alone, but rather a process that reflects access to the mental lexicon.

As for the N400 following the PMN, it has been reported that when both contextual and lexical information for a word is available, the contextual information influences the N400, decreasing its amplitude in words that match the context (van Berkum et al., 1999). Therefore, Kutas and Federmeier (2000) describe the N400 as reflecting “integration into the context.” Deacon et al. (2004) stated that it is generated by orthographic/phonological analysis and is affected by semantic information in a “top-down” manner. In this study, in the existing word task, words are presented in both the match and mismatch conditions, and match/mismatch judgments must be integrated into the context of the pictures. Therefore, the difference in N400 between the match and mismatch conditions in this task may reflect not only access to the mental lexicon, but also semantic processing (integration into the context). The results obtained in this study give clues as to what processes PMN and N400 reflect: PMN may reflect the phonological and lexical processing, while N400 may reflect a higher-order processing, the process of integration into context.

As a word is being presented auditorily, several candidate words (cohort) that start with the same phoneme are activated in the listener’s mental lexicon. As the phonemes are perceived one at a time, the candidate vocabulary is reduced to the final pertinent term (Marslen-Wilson, 1987). This process has also been supported by brain wave evaluation, which discovered that for the semantic processing of vocabulary, the responses to the phoneme of the words change over time. In the McMurray et al. (2022) experiment, after target words were presented auditorily, a word phonologically similar (cohort) to the target word, and a word unrelated to the target word was presented in written form. The task was to choose which one is closer to the phonetically presented word. The results showed a characteristic pattern in which the target word and its phonologically similar competitor word (cohort) are active immediately after word onset (at levels that were greater than unrelated items), and at 100 ms activation levels were the same. However, 500 ms after word presentation, the response to the competing cohort is suppressed and the target is selected. If we interpret the findings of this study through the lens of the processing of words presented auditorily, PMN seems to reflect the activation of word candidates (cohorts) from the point of speech input and the process of lexical judgments in the search for the corresponding word. The subsequent N400 reflects the higher-level semantic processing of contextual integration.

Although sound symbolic pseudowords are non-words, both PMN and N400 increased in amplitude in the mismatch condition relative to the matching condition, same as in the case of existing sound symbolic words. This suggests that even though sound symbolic pseudowords are non-words, they are processed similarly to standard words in terms of semantics. Access to meaning through automatic activation of phonological information has been reported for non-words with phonological features similar to those of real words (Kujala et al., 2004). The results of this study show that this is also the case for physiological brain responses. This experiment included a situation picture and word/pseudoword matching task. The higher processing effort required for the mismatch condition compared to the match condition for the sound-symbol pseudowords suggests that word-level processing, such as retrieving word candidates, was also performed for non-word sound symbolic pseudowords. Furthermore, the increase in N400 amplitude in the mismatch condition for the sound symbolic pseudowords is consistent with Deacon et al. (2004). Therefore, it has been suggested that the sound-symbolic pseudowords are indicative of processing at a higher level, that is, semantic processing, including integration into the context. In the difference between the match and mismatch conditions for sound symbol pseudowords indicates that sound symbol pseudowords are not judged on a simple impression level (such as spiky/round) or activated by other words with similar spellings (phonological structures) in the lexicon, but are judged on the similar context basis as actual words.

Language is the process of ascribing a label to a situation or object. It emerges as a result of semantic integration of auditory and visual information. Attentional processes are also involved (Wang et al., 2017; McCormick et al., 2018). Multiple integration processes in various cortical areas are involved in semantic integration, which is modulated by attention (Xi et al., 2019). According to McCormick et al. (2021), greater attention may be needed for the adjusting and controlling of information processing for discrepancies between visual and auditory information when attention is focused on auditory information. In this task, the concept that a word meant was presented as an illustration, and if the word matched the presented illustration, it was processed quickly and with low processing load due to the top-down effect of context. However, if the word did not match, the participants had to reinterpret the picture and search again for a possible match with the scene/event, which would have required more attention due to the processing load of adapting and suppressing information processing. As shown in Figure 1, in addition to representing the softness of the dog’s fur, the picture could also represent the act of touching the dog or the feelings of the person touching the dog. It has been noted that higher-level processing of elements like syntax and contextual meaning is also carried out during the semantic processing of a word as sounds are entered one at a time and the candidate vocabulary is narrowed down; thus contextual-level semantic information has a significant impact on semantic processing (Dahan and Tanenhaus, 2004; Costa et al., 2009; McMurray et al., 2022). We assume that in the existing word task and pseudoword task, the increased amplitude and latency delay in the mismatch condition may have reflected the search for alternative interpretation of what the stimulus picture may represent, the top-down influence of different possibilities, and the load in narrowing the vocabulary. In addition, the present results showed that the amplitude of both the PMN and the N400 was increased for sound-symbolic pseudowords (non-words) compared to sound-symbolic words (words). Sound-symbolic pseudowords are not words that are stored in the mental lexicon. Therefore, the expectation of this result was that sound-symbolic pseudowords would require more extensive processing (attention). In this study, we were able to show the differences between the semantic processing of existing sound symbolic words and pseudowords in terms of the physiological responses of the brain. The similarities between sound symbolic pseudowords and conventional words suggest that both are processed temporally from the input of phonemes to the contextual semantic processing. The difference was that the processing load for sound symbolic pseudowords was greater than that for sound symbolic words. In order to clarify this difference, it will be necessary to identify the brain regions involved in the semantic processing of both sound symbolic words and pseudowords, which is an issue for the future. Although fMRI at high spatial resolution or other methods could be used to search for the region of the brain responsible for semantic processing of sound symbolic pseudowords, it is challenging to distinguish PMN from N400 due to inferior temporal resolution. Therefore, a more detailed analysis using a combination of various neuroimaging techniques is needed. In addition, Japanese is a language that is rich in onomatopoeia and ideophones and commonly makes use of sound symbolism. This could be why sound symbolic words are processed is a way similar to the semantic processing of other words of the language. Thus, a cross-linguistic approach is necessary for future studies in order to clarify the semantic processing of sound symbolism.

## 5. Conclusion

In this study, the semantic processing of sound symbolic words included in the mental lexicon is compared to the semantic processing of sound symbolic pseudowords, which have no concrete meaning stored in the lexicon, but use sounds that are consistently associated with a certain meaning. It also clarifies the processes reflected by PMN and N400, which are used in many studies of lexical semantic processing. The findings of this study suggest that PMNs may reflect not only phonological processing, but also further processing up to the point of accessing vocabulary, whereas N400 may reflect final semantic judgments. Furthermore, similar responses toward sound symbolic words and pseudowords were observed in the match and mismatch conditions, indicating that both of them undergo semantic processing.

## Data availability statement

The original contributions presented in this study are included in the article/Supplementary material, further inquiries can be directed to the corresponding author.

## Ethics statement

The studies involving humans were approved by the International University of Health and Welfare’s Ethical Review Committee. The studies were conducted in accordance with the local legislation and institutional requirements. The participants provided their written informed consent to participate in this study.

## Author contributions

KS and HO contributed to conception and design of the study. KS and SK performed the experiments, collected the data, and performed the statistical analysis. KS wrote the first draft of the manuscript. KS, SK, JI, MP and HO wrote sections of the manuscript. MP edited English language. All authors contributed to manuscript revision, read, and approved the submitted version.

## Footnotes

1
https://www.audacityteam.org
