# Supplementary material for: Cognitive neural responses in the semantic comprehension of sound symbolic words and pseudowords
Source: Front Hum Neurosci. 2023 Oct 11;17:1208572. doi: 10.3389/fnhum.2023.1208572 (PMC10603230; doi:10.3389/fnhum.2023.1208572)
Supplement: Supplementary file 1 [file Data_Sheet_1.pdf]

Supplementary Material

Cognitive Neural Responses in the Semantic Comprehension of Sound Symbolic Words and Pseudowords

Kaori Sasaki*, Seiichi Kadowaki, Junya Iwasaki, Marta Pijanowska, Hidehiko Okamoto

*** Correspondence:** Kaori Sasaki: kaori.s@iuhw.ac.jp

1 Supplementary Data

Sound Symbolic words		sound symbolic pseudowords	
match	mismatch	match	mismatch
zuruzuru	punipuni	zusuzusu	pubipubi
gayagaya	jorijori	gawagawa	jojijoji
mishimishi	jitojito	mikimiki	jinojino
bukubuku	zukizuki	bufubufu	zuchizuchi
giigii	nukunuku	gikigiki	nufunufu
katakata	chikuchiku	kadakada	chipuchipu
gutsugutsu	zarazara	gufugufu	zasazasa
korokoro	nebaneba	konokono	nepanepa
bachabacha	mokomoko	bazabaza	mohomoho
garagara	pokapoka	ganagana	pohapoha
dosadosa	muzumuzu	dozadoza	mugumugu
chokichoki	sarasara	chochichochi	sanasana
patapata	nechanecha	pahapaha	nenyanenya
jarajara	nyurunyuru	jadajada	nyununyunu
gachagacha	jinjin	gakyagakya	jishijishi
jabujabu	funyafunya	jafujafu	furyafurya
chorochoro	fuwafuwa	chopochopo	fuhafuha
poripori	igaiga	pogipogi	idaida
gunyagunya	zokuzoku	guryagurya	zozuzozu
kasakasa	jimejime	kazakaza	jinejine
chikuchiku	katakata	chipuchipu	kadakada
jorijori	gayagaya	jojijoji	gawagawa
mokomoko	bachabacha	mohomoho	bazabaza
sarasara	chokichoki	sanasana	chochichochi
punipuni	zuruzuru	pubipubi	zusuzusu
jinjin	gachagacha	jishijishi	gakyagakya
zokuzoku	gunyagunya	zozuzozu	guryagurya

zukizuki	bukubuku	zuchizuchi	bufubufu
pokapoka	garagara	pohapoha	ganagana
nebaneba	korokoro	nepanepa	konokono
igaiga	poripori	idaida	pogipogi
jitojito	mishimishi	jinojino	mikimiki
nukunuku	giigii	nufunufu	gikigiki
jimejime	kasakasa	jinejine	kazakaza
fuwafuwa	chorochoro	fuhafuha	chopochopo
funyafunya	jabujabu	furyafurya	jafujafu
nechanecha	patapata	nenyanenya	pahapaha
muzumuzu	dosadosa	mugumugu	dozadoza
nyurunyuru	jarajara	nyununyunu	jadajada
zarazara	gutsugutsu	zasazasa	gufugufu
