# Supplementary material for: Cognitive neural responses in the semantic comprehension of sound symbolic words and pseudowords
Source: Front Hum Neurosci. 2023 Oct 11;17:1208572. doi: 10.3389/fnhum.2023.1208572 (PMC10603230; doi:10.3389/fnhum.2023.1208572)
Supplement: Supplementary file 2 [file Data_Sheet_2.pdf]

word vs. pseudoword	Sound symbolic pseudoword		Sound symbolic word	
match vs. mismatch	match	mismatch	match	mismatch
N-1	-2.2912	-2.1334	1.8912	-2.7651
N-2	3.6116	-1.4248	7.0915	5.5737
N-3	-4.0308	-4.4723	-2.2739	-4.0896
N-4	0.3415	0.4369	-1.6858	-1.2876
N-5	3.3684	-0.8505	8.3979	1.1739
N-6	3.5036	0.2068	4.0557	-0.5091
N-7	1.5411	-3.7881	0.3446	-2.7893
N-8	-2.5972	-3.5315	-2.6975	-1.7308
N-9	0.8139	1.2944	2.8228	0.4573
N-10	1.168	0.323	1.0526	1.7257
N-11	2.7516	3.6168	2.218	3.5408
N-12	-9.0675	-9.6775	-4.6887	-7.7021
N-13	-6.0561	-12.2925	-2.1554	-7.5277
N-14	-4.8261	-2.8396	1.0517	-0.4541
N-15	-13.4096	-14.5823	-14.1891	-13.7815
N-16	-1.9714	-4.667	-0.764	0.1178
N-17	-7.0184	-5.8735	-4.2278	-4.5689
