# Supplementary material for: Cognitive neural responses in the semantic comprehension of sound symbolic words and pseudowords
Source: Front Hum Neurosci. 2023 Oct 11;17:1208572. doi: 10.3389/fnhum.2023.1208572 (PMC10603230; doi:10.3389/fnhum.2023.1208572)
Supplement: Supplementary file 3 [file Data_Sheet_3.pdf]

word vs. pseudoword	Sound symbolic pseudoword		Sound symbolic word	
match vs. mismatch	match	mismatch	match	mismatch
N-1	366	379	351	353
N-2	386	396	391	403
N-3	382	382	390	381
N-4	359	360	375	389
N-5	395	403	397	414
N-6	372	407	363	401
N-7	383	397	363	408
N-8	382	368	374	392
N-9	436	451	379	362
N-10	403	397	393	391
N-11	341	327	348	382
N-12	355	409	400	405
N-13	374	378	384	401
N-14	416	420	366	399
N-15	348	368	368	395
N-16	351	385	359	369
N-17	367	376	366	374
