# Supplementary material for: Cognitive neural responses in the semantic comprehension of sound symbolic words and pseudowords
Source: Front Hum Neurosci. 2023 Oct 11;17:1208572. doi: 10.3389/fnhum.2023.1208572 (PMC10603230; doi:10.3389/fnhum.2023.1208572)
Supplement: Supplementary file 4 [file Data_Sheet_4.pdf]

word vs. pseudoword	Sound symbolic pseudoword		Sound symbolic word	
match vs. mismatch	match	mismatch	match	mismatch
N-1	-1.861	-2.849	2.653	-1.933
N-2	2.355	-1.070	7.517	6.467
N-3	2.311	1.577	5.443	0.929
N-4	-0.246	-0.480	-0.065	-0.480
N-5	4.853	0.939	7.939	3.046
N-6	1.719	0.202	3.550	0.187
N-7	3.149	-2.345	1.334	-0.671
N-8	0.973	-4.183	2.938	-1.000
N-9	0.336	1.301	2.684	-3.173
N-10	1.278	1.200	3.304	2.669
N-11	3.733	6.068	5.444	4.976
N-12	-8.052	-9.465	-0.347	-4.485
N-13	-2.936	-9.434	-0.251	-2.221
N-14	-3.053	-1.380	2.483	0.129
N-15	-10.826	-12.127	-10.121	-12.605
N-16	-1.341	-7.466	-0.994	-0.590
N-17	-0.709	-1.119	1.557	-1.519
